# Speman^®^, A Proprietary Ayurvedic Formulation, Reverses Cyclophosphamide-Induced Oligospermia In Rats

**DOI:** 10.5195/cajgh.2013.14

**Published:** 2013-04-02

**Authors:** Mohd. Azeemuddin Mukram, Mohamed Rafiq, Suryakant D. Anturlikar, Pralhad S. Patki

**Affiliations:** 1 Department of Pharmacology, R&D Center, The Himalaya Drug Company, Makali, Bangalore, India

**Keywords:** Speman^®^, Cyclophosphamide, Oligospermia, Sperm count

## Abstract

**Background:**

This investigation was aimed to evaluate the effect of Speman^®^, a well known ayurvedic proprietary preparation, in an experimental model of cyclophosphamide-(CP) induced oligospermia in rats.

**Materials and Methods:**

Thirty male rats were randomized in to five, equally-sized groups. Rats in group 1 served as a normal control; group 2 served as an untreated positive control; groups 3, 4, 5 received Speman^®^ granules at doses of 300, 600, and 900mg/kg body weight p.o. respectively, once daily for 13 days. On day four, one hour after the respective treatment, oligospermia was induced by administering a single dose of CP (100mg/kg body weight p.o.) to all the groups except group1. At the end of the study period the rats were euthanised and accessory reproductive organs were weighed and subjected to histopathological examination. The semen samples were subject to enumeration of sperms. Weight of the reproductive organs, histopathological examination of the tissues, and sperm count were the parameters studied to understand the effect of Speman^®^ on rats with CP-induced oligospermia.

**Results:**

Changes that occurred due to the administration of CP at a dose of 100 mg/kg body weight were dose dependently reversed with Speman^®^ at a dose of 300, 600, and 900 mg/kg body weight. There was a statistically significant increase in sperm count and the weight of the seminal vesicle, epididymis, and prostate.

**Conclusion:**

Findings of this investigation indicate that Speman^®^ dose dependently reversed the CP-induced derangement of various parameters pertaining to the reproductive system. This could explain the total beneficial actions of Speman^®^ reported in several other clinical trials.

## Introduction

Significant growth of the global human population over the years has been paralleled by a substantial increase in the number of infertile couples, a major reproductive-health concern owing to the fact that it affects quality of human life. In human patients, stresses and strains of modern living may make an individual sexually neurasthenic and functionally impotent. This may lead to a chain of psychological complexes, contributing to sexual inferiority and its allied syndromes. Infertility affects both men and women. Current epidemiological evidence suggests that 15% of couples in the world experience infertility, half of which remain untreated and/or unresolved. Among infertile couples, 40% are primarily due to the infertility of the male partner, while 20% of these cases are a combination of both male and female factors.^1^ Oligospermia, asthenospermia, teratozoospermia, and azoospermia account for 20% to 25% of male infertility cases. Clinically, oligospermia (sperm concentrations of < 20 million sperms/mL of semen) is considered to be one of the most prevalent causes of male infertility.^2^

Though the pathophysiology of oligospermia remains unclear, hormones like follicular stimulating hormone (FSH) and luteinizing hormone (LH), which play an important role in spermatogenesis, act as marker components^3^. Treatment options for oligospermia in modern medicine are still in their infancy given that no specific drug has yet been discovered. In the last few years, extensive research has been carried out in the field of Ayurveda for utilizing the natural sources in the treatment of oligospermia.

Speman^®^, a polyherbal proprietary formulation developed by The Himalaya Drug Company (Makali, Bangalore) is approved by the Government of India’s Drug Regulatory Authority (Department of Ayush, Ministry of Health and Family Welfare). It is used clinically in the management of male sexual disorders like oligospermia, premature ejaculation, senile sex aberrations, and several other related conditions.^4^–^5^ Several reports are available indicating the beneficial effects of a Speman^®^, on the gametogenic as well as androgenic functions of the testes in humans and animals.^6^–^11^

Speman^®^ constitutes a mixture of extracts of *Withania somnifera*, *Asteracantha longifolia*, *Lactuca scariola*, *Mucuna pruriens*, *Parmelia parlata, Argyreia speciosa*, *Tribulus terrestris*, *Leptadenia reticulate, and* Suvarnavang. The formulation has been tested for its quality and consistency at every step of the manufacturing process as per the accepted principles of Good Manufacturing Practice (GMP) and Good Laboratory Practice (GLP). Its botanical identification, quality parameters, and ayurvedic criteria comply with the international guidelines and pharmacopoeial standards.

## Materials and Methods

This investigation was discussed and approved by the Institutional Animal Ethics Committee (IAEC) of The Himalaya Drug Company, Bangalore, on 4/01/2008 *vide* Protocol No. 83.

Its aim was to evaluate the effect of Speman^®^ in an experimental model of CP-induced oligospermia in rats.

### Drugs and chemicals

Speman^®^ (The Himalaya Drug Company, Makali, Bangalore), Cyclophosphamide (CP) (German Remedies Limited, Mumbai, India), and all the other chemicals used in the experiments were of analytical grade and purchased from reputed suppliers.

### Experimental animals

Inbred Wistar rats weighing 250–300 g were used for the study; they were housed under standard conditions of temperature (22 ± 3°C), relative humidity (55 ± 5%), and light (12 h light/dark cycle) before and during the study. They were fed with a standard pellet diet and water *ad libitum*. All animals received humane care as per the guidelines prescribed by the Committee for the Purpose of Control and Supervision of Experiments on Animals (CPCSEA), Ministry of Environment & Forests, Government of India.

## Experimental protocol

Thirty male rats were randomized into five groups of six rats each. Animals from group 1 and 2 received water once a day, orally (p.o.), at a dose of 10 mL/kg body weight; and served as the normal and positive controls, respectively, while those in groups 3 to 5 received Speman^®^ granules at a dose of 300, 600, and 900 mg/kg body weight p.o. once daily for 13 days. On day four, one hour after their respective treatments, animals in all groups except group 1 received CP at a single dose of 100 mg/kg body weight p.o. Treatment with Speman^®^ was continued for 9 days after administration of CP. At the end of treatment period, on day-14, the animals were euthanized and accessory reproductive organs such as the testes, epididymis, and seminal vesicle were collected, weighed, fixed in neutral buffered formalin solution, and sent for routine histopathological examinations. Semen samples from unilateral cauda were subjected to enumeration of sperms following dilution with sperm diluting fluid. Counting was performed using a haemocytometer and light microscope with 100× magnification.^12^, ^13^

## Results

This preclinical study was carried out to evaluate the effect of Speman^®^ in an experimental model of CP-induced oligospermia in rats. Its efficacy was tested at dose levels of 300, 600, and 900 mg/kg body weight p.o., which corresponds to or equivalent to the recommended human dose. Dosing volume was decided based on the mean body weight of the animals in each group. Administration of CP (100 mg/kg p.o.) to rats induced significant changes, like oligospermia, weight reduction of seminal vesicle, epididymis, and testes.

Sperm count of the normal control group was 46.3 ± 2.89 million/mL of semen and that of the untreated positive control (CP-induced group) was 30.7 ± 1.13 million/mL of semen. The decrease in sperm count observed after administration of CP was reversed dose-dependently by treating with different doses of Speman^®^ (300, 600, and 900 mg/kg). The sperm count was 36.9 ± 1.39, 40.6 ± 0.72, and 42.4 ± 1.07 million/mL in animals from groups 3, 4, and 5, respectively, illustrated in:

**Figure 1:**
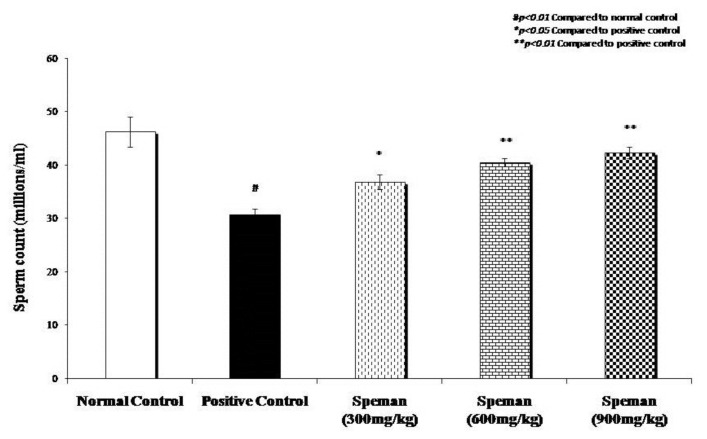
Effect of Speman^®^ on sperm count in an experimental model of CP-induced oligospermia in rats.

## Increase in sperm count was found to be statistically significant compared to the positive control

**Figure 2:**
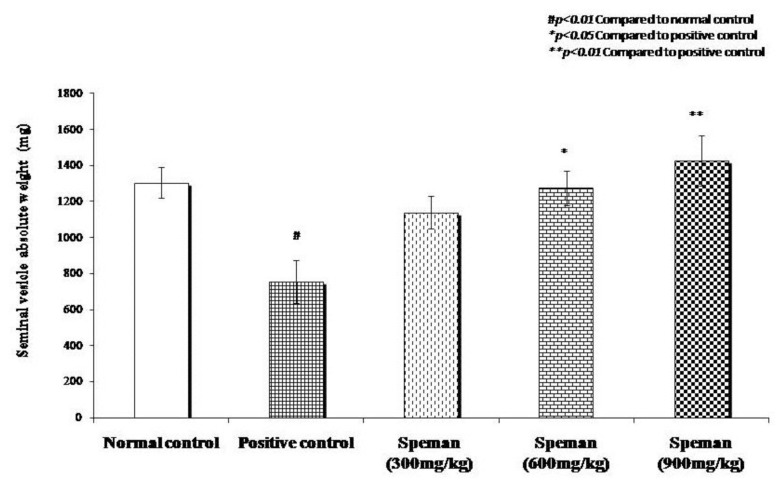
Effect of Speman^®^ on the absolute weight of seminal vesicle in an experimental model of CP-induced oligospermia in rats.

**Figure 3:**
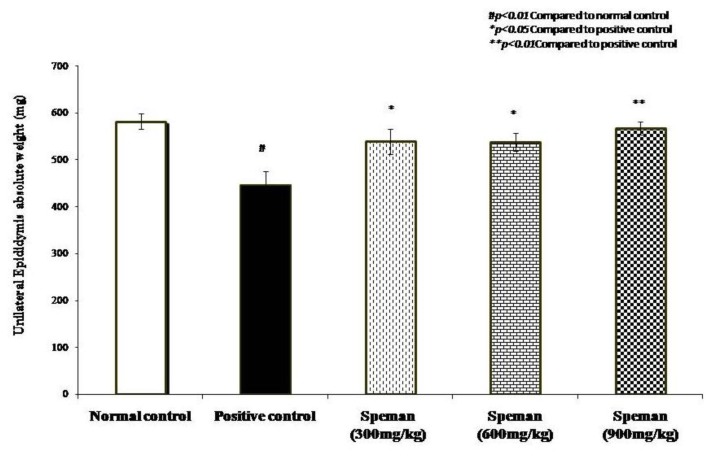
Effect of Speman^®^ on the absolute weight of unilateral epididymis in an experimental model of CP-induced oligospermia in rats.

**Figure 4:**
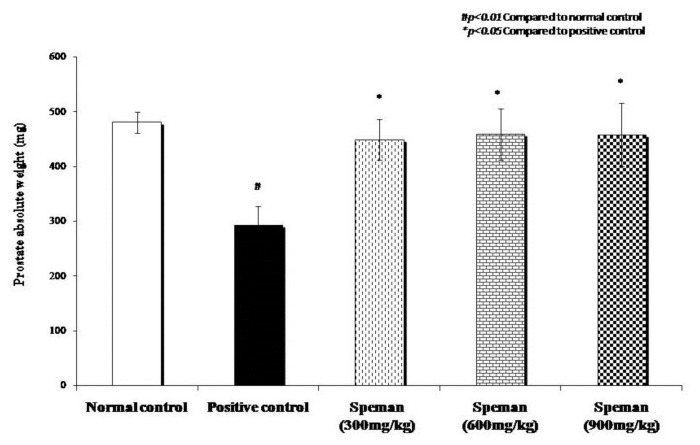
Effect of Speman^®^ on absolute weight of prostate in an experimental model of CP-induced oligospermia in rats.

**Figure 5:**
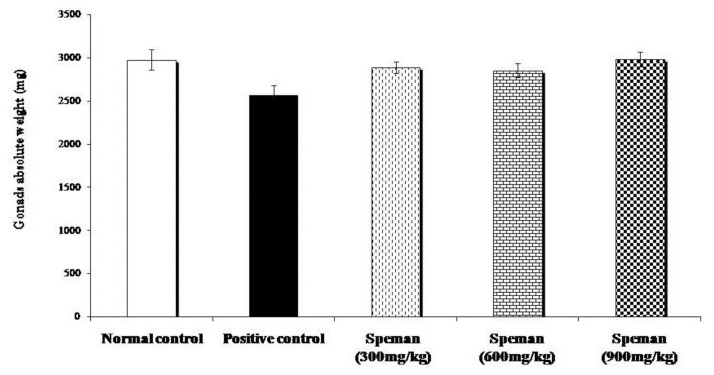
Effect of Speman^®^ on the absolute weight of gonads in an experimental model of CP-induced oligospermia in rats.

Decrease in weight of seminal vesicles, unilateral epididymis, prostate, and gonads due to the administration of CP was reversed dose-dependently by treating with different doses of Speman^®^. Increase in weight in all tested doses was found to be statistically significant compared to the positive control group (Figures 2–5, links below).

The histopathological report (see Table 1) states that treatment with Speman^®^ dose-dependently decreased the mean lesion score of testes, prostate, epdidymis, and seminal vesicles.

**Table 1:** Results of the histopathological evaluation of the accessory reproductive organs following Speman^®^ treatment in rats with CP-induced oligospermia

Organs	Lesions	Normal control	Positive control	Speman^®^ (300 mg/kg)	Speman^®^ (600 mg/kg)	Speman^®^ (900 mg/kg)
Testes	Interstitial oedema	1.67 ± 0.21	2 ± 0.63	2 ± 0	1.5 ± 0.22	2.4 ± 0.24
	Tubular degeneration	-	3.17 ± 0.31	3 ± 0	1.6 ± 0.25	2.2 ± 0.66
Prostate	Epithelial thinning	-	4 ± 0	3 ± 0.52	1.8 ± 0.37	1.25 ± 0.32
Epididymis	Epithelial proliferation	-	1.83 ± 0.48	1.67 ± 0.21	1.5 ± 0.22	1.0 ± 0
Seminal vesicle	Degeneration		2 ± 0.32	1.5 ± 0.29	2 ± 0.58	2 ± 0.52

*Severity score*: Minimal: 1 (very small amount of changes ? 10%); Mild: 2 (lesion is easily identified with limited severity 11%–25%); Moderate: 3 (lesion is predominant 26%–75%); Severe: 4 (degree of changes is 76%–100%, that is, great enough in intensity or extent to expect significant tissue or organ dysfunction.

## Discussion

In the present study, CP was used to induce oligospermia in rats. CP is a cytotoxic alkylating agent used in the treatment of neoplasia and autoimmune disorders. Its cytotoxic effects are the result of chemically-reactive metabolites that alkylate DNA and proteins, producing cross-links.^14^ It affects rapidly-dividing cells and damages the highly proliferative testis. Thus, the use of this drug for treatment of neoplasia in male patients increases the incidence of oligospermia and azoospermia resulting in male infertility.^15^ Animal studies have revealed that treatment of rats/mice with CP leads to transitory oligospermia, decreased testicular weight, DNA synthesis in spermatogonia, protein synthesis in spermatids, and biochemical and histological alterations in the testis and epididymis.^16^–^18^ The precise mechanism by which CP causes testicular toxicity is unknown. However, numerous studies have shown that exposure to CP can disrupt the redox balance of tissues, suggesting that biochemical and physiological disturbances may result from oxidative stress.^19^–^21^

The beneficial effects of Speman^®^ granules in CP-induced oligospermia are due to the synergistic action of the various herbs used in the formulation of Speman^®^. Most of the herbs present in this formulation have been reported to have a beneficial effect on the male reproductive organs—*Mucuna pruriens* reduces stress and improves the quality of semen in infertile men^22^; *Argyreia nervosa* is known for its aphrodisiac property^23^; *Asteracantha longifolia* and *Hygrophila spinosa* are reported to improve sexual behavior and reproductive function in male rats^24^,^25^; *Tribulus terrestris* ameliorates the testicular development of immature albino rats.^26^

## Conclusion

From the findings reported here, it can be concluded that Speman^®^ is effective in increasing sperm count and weight of sex organs like gonads, seminal vesicles, prostate, and epididymis in rats with CP-induced oligospermia. Treatment with Speman^®^ showed dose-dependent and significant reversal of the CP-induced changes under the testing conditions and doses employed.
